# Ageing-Associated Transcriptomic Alterations in Peri-Implantitis Pathology: A Bioinformatic Study

**DOI:** 10.1155/2022/8456968

**Published:** 2022-10-11

**Authors:** Zhaojun Tian

**Affiliations:** College of Dentistry, I.M. Sechenov First Moscow State Medical University, Bolshaya Pirogovskaya Street, No. 2с4, Moscow 119435, Russia

## Abstract

**Background:**

Ageing is associated with increased incidence of peri-implantitis but the roles of ageing-associated biological mechanisms in the occurrence of peri-implantitis are not known. This study is aimed at performing integrative bioinformatic analysis of publically available datasets to uncover molecular mechanisms related to ageing and peri-implantitis.

**Methods:**

Gene expression datasets related to ageing and peri-implantitis (PI) were sought, and differentially expressed genes (DEGs) were analysed. Ageing-related genes were also identified from the “Aging Atlas” database. Using intersection analysis, an age-related-PI gene set was identified. Functional enrichment analysis for enriched GO biological process and KEGG pathways, protein-protein interaction (PPI) network analysis, correlation analysis, and immune cell infiltration analysis to determine high-abundance immune cells were performed. Least absolute shrinkage and selection operator (LASSO) logistic regression identified key age-related-PI genes. Transcription factor-gene and drug-gene interactions and enriched KEGG pathways for the key age-related-PI genes were determined.

**Results:**

A total of 52 genes were identified as age-related-PI genes and found enriched in several inflammation-associated processes including myeloid leukocyte activation, acute inflammatory response, mononuclear cell differentiation, B cell activation, NF-kappa B signalling, IL-17 signalling, and TNF signalling. LYN, CDKN2A, MAPT, BTK, and PRKCB were hub genes in the PPI network. Immune cell infiltration analysis showed activated dendritic cells, central memory CD4 T cells, immature dendritic cells, and plasmacytoid dendritic cells were highly abundant in PI and ageing. 7 key age-related PI genes including ALOX5AP, EAF2, FAM46C, GZMK, MAPT, RGS1, and SOSTDC1 were identified using LASSO with high predictive values and found to be enriched in multiple neurodegeneration-associated pathways, MAPK signalling, and Fc epsilon RI signalling. MAPT and ALOX5AP were associated with multiple drugs and transcription factors and interacted with other age-related genes to regulate multiple biological pathways.

**Conclusion:**

A suite of bioinformatics analysis identified a 7-signature gene set highly relevant to cooccurrence of ageing and peri-implantitis and highlighted the role of neurodegeneration, autoimmune, and inflammation related pathways. MAPT and ALOX5AP were identified as key candidate target genes for clinical translation.

## 1. Introduction

Dental implants have emerged as a widely practised globally [1] and predictable modality for replacement of missing teeth [2] with high success rates [3]. Dental implant technology is also rapidly evolving to enable improved treatment outcomes [4]. Loss of teeth increases with age [5], and thus, a high proportion of patients who require tooth replacement and receive dental implants are elderly [6]. While dental implants show high treatment success rates in seniors [7], increased rates of biological complications including peri-implantitis [8] and implant failure [9] among elderly are also reported.

Peri-implant diseases include peri-implant mucositis and peri-implantitis, which comprises a plaque biofilm-associated inflammatory destruction of implant supporting bone, marked by bone-loss, bleeding on probing and peri-implant pocket formation, analogous to periodontitis [10], and is a chief cause of implant failure [11] or severe complications such as osteonecrosis in susceptible patients [12]. As with periodontitis [13], peri-implantitis is found to occur at higher rates among elderly individuals [8, 14]. However, the two diseases have important differences owing to the difference in the nature of tissues surrounding the dental implant, including a weaker soft-tissue barrier at the implant-mucosa interface [13]. Furthermore, the treatment of peri-implantitis has less predictable outcomes as compared to periodontitis [15, 16], which necessitates a greater understanding of underlying disease mechanisms. Molecular mechanisms underlying peri-implantitis pathology have been researched [17]. Elderly patients may be more likely to have known risk factors and indicators for peri-implantitis such as diabetes mellitus, osteoporosis, or certain medications [7], which could account for higher rates of peri-implantitis. The ageing process is associated with several alterations in tissue and cellular turnover including “immunosenescence” [18]. Age greater than 65 years has been independently associated with peri-implantitis in multiple studies [8, 14, 19]. It can be hypothesised that ageing-related cellular processes might also contribute to increased incidence of peri-implant disease independently from other known risk factors. At present, little is known about ageing-related molecular processes and gene-expression patterns that may be linked to peri-implantitis.

Therefore, the present investigation aimed to uncover ageing related transcriptomic changes that could be candidate mechanisms functional in peri-implantitis and comprehensively investigate related molecular mechanisms including biological pathways, transcription factors, and pharmacological agents. These data can provide important insights into ageing related biological pathways that may predispose elderly patients to peri-implant diseases. Such findings could uncover experimental research directions for clinical translation in biomaterial design and therapeutics to prevent and intercept peri-implant diseases among elderly patients.

## 2. Material and Method

### 2.1. Datasets

We downloaded the peri-implantitis (PI) microarray datasets GSE33774 [17] and GSE106090 [20] from the GEO database (http://www.ncbi.nlm.nih.gov/) and selected PI-related samples. Next, we downloaded ageing-related gene expression datasets GSE83382 and GSE180588 [21]. We selected gingival tissue samples associated with ageing, where the GSE83382 comprised high throughput sequencing data and the GSE180588 comprised microarray data. The datasets used for this analysis are shown in Table 1. We further download ageing-related genes from the database “Aging Atlas” (https://ngdc.cncb.ac.cn/aging/age_related_genes).

### 2.2. Data Preprocessing

If the downloaded dataset type was an array, we converted the probe id to gene symbol based on the platform information corresponding to the dataset. If the dataset type was high throughput sequencing, we first downloaded the annotation file from GENCODE (https://www.gencodegenes.org/human/) and then obtained the mapping information for the gene symbol and probe ID from the dataset corresponding platform. We used the annotation file to map the probe ID to the gene symbol, and we performed the conversion of the probe ID to the gene symbol. When one probe ID matched multiple gene symbols, we performed deduplication of the gene symbol with the mean of the sample expression value and finally obtained the transformed expression matrix. After obtaining the gene symbol expression matrix, we performed log2 conversion (log2) for the datasets with large sample expression values (GSE33774 and GSE180588). When the expression value of a gene was 0 in more than half of the samples, then, we removed that gene from the expression matrix.

### 2.3. Differential Gene Expression Analysis

We used the “*limma*” R package (R version 4.1.3) to perform differential gene expression analysis of the PI datasets and the ageing datasets, with the comparison method case vs. control. To obtain potential ageing-related PI genes, we selected differentially expressed genes (DEG) based on the results of each dataset's analysis. For GSE33774, GSE180588, and GSE83382, we selected the genes of *p* value < 0.05 and |log (fold change) | > 0.5 as differentially expressed genes. For GSE106090, we selected the genes *p* < 0.05 and |log (fold change)| > 1.5 as DEG.

### 2.4. Prediction of Ageing-Related PI Genes

The genes that were significantly upregulated or downregulated in the 2 sets of PI datasets were considered PI-DEGs. The genes that were up- or downregulated in the ageing datasets were considered as ageing-DEGs. Next, we extracted the intersection of these two sets, which were considered potential ageing-related PI-genes. We labelled these genes age-related PI gene set 1. In addition, we intersected PI DEGs and ageing-related genes obtained from the Ageing Atlas database and labelled these genes as age-related PI gene set 2. Finally, we merged age-related PI gene set 1 and age-related PI gene set 2 to obtain the final age-related PI gene set. These genes were significantly expressed in peri-implantitis and are also closely related to ageing.

### 2.5. Functional Enrichment for Age-Related PI Genes

In order to observe the gene expression of age-related PI genes in different samples, we extracted the expression values of age-related PI genes in the PI and age datasets and then used the “*pheat map*” in R to draw a heat map for display. The “ClusterProfiler” package in R was then used to analyse the biological process and biological pathways enriched in these genes.

### 2.6. PPI Network for Age-Related PI Genes

We obtained the protein-protein interaction (PPI) relationship pairs between the age-related PI genes and other genes from the HPRD database (http://www.hprd.org/) and the BIOGRID (http://thebiogrid.org/) database. We merged the PPI data obtained from the two databases and built a PPI network using Cytoscape (version 3.8). We mapped other ageing-related genes in the Ageing Atlas database into the network, analysing the relationship with age-related PI gene and other ageing-related genes in the biological network. We analysed the nature of the network topology using the cyberscape plug-in network analyzer after the network was built. Finally, we filtered the hub nodes according to the nature of the topology.

### 2.7. Correlation Analysis between Age-Related PI Gene

To analyse the correlation among age-related PI genes in PI, we combined case and control samples from the two sets in PI and then obtained the expression values of age-related PI genes in the samples. Finally, we used the Pearson correlation coefficient to see the relationship between age-related PI genes over different sample types.

### 2.8. Immune Cell Infiltration Analysis for PI

A total of 782 immune genes and 28 immune cell types were obtained from the literature [22] to obtain a total of 782 immune genes and 28 immune cell types. In order to analyse the relationship between genes in PI and immune cells, we first obtained the expression matrices of GSE33774 and GSE106090 and then quantified their GSVA packets using R. With ssGSEA analysis, we obtained the cell abundance of immune cells in the two data sets' samples. We then combined the results of the two sets to obtain immune cells with relatively high cell abundance in both datasets by clustering at the case sample level. We used the Pearson correlation coefficient to analyse the correlation between immune cells and analyse the differences in the abundance between normal and disease samples using a violin plot. We also used the Wilcoxon test to test differences between the two sets of samples, while analysing differences in case sample-related immune cell abundance in GSE33774 and GSE106090.

In addition, we also performed ssGSEA quantitative analysis of case samples from the ageing datasets GSE83382 and GSE180588 and finally obtained immune cells with relatively high cell abundance in both datasets through hierarchical clustering analysis. Finally, we obtained high-abundance immune cells in both PI and age.

### 2.9. Further Screening for Age-Related PI Genes

To further acquire the important age-related PI genes, we first extracted the gene expression values of age-related PI genes in the two datasets of PI. We then performed ANOVA analysis based on sample type (case and control) to obtain the age-related PI genes (*p* value < 0.05) that were significant in both datasets. We then used least absolute shrinkage and selection operator (LASSO) logistic regression to screen the significant age-related PI genes. We first merged the two datasets of PI and then extracted the expression values of significant age-related PI genes. We used LASSO to build a model for feature screening according to the sample type. We constructed a penalty function to obtain a more refined LASSO model to achieve the selection of key genes. The genes obtained by the LASSO analysis can be considered to play important roles in PI and ageing, which we labelled as key age-related PI genes. We obtained the expression values of key age-related PI genes in the two datasets of PI and then used ROC analysis to predict the effect of hub genes.

### 2.10. Relationship between High-Abundance Immune Cells and Key Age-Related PI Gene

We obtained the fraction of high-abundance immune cells and the expression of key age-related PI genes in the case samples of PI. Based on the fraction of high-abundance immune cells, the expression of key age-related PI genes, and Pearson correlation coefficient, we analysed the correlation between the high-abundance immune cells and the key age-related PI genes.

### 2.11. Key Age-Related PI Gene, Transcription Factor, and Drug Relationships

We downloaded the transcription factor (TF) and key age-related PI gene pairs from TRRUST (https://www.grnpedia.org/trrust/), cGRNB (https://www.scbit.org/cgrnb), HTRIdb (http://www.lbbc.ibb.unesp.br/htri/), ORTI (http://orti.sydney.edu.au/about.html), and TRANSFAC (http://gene-regulation.com/pub/databases.html). To analyse the relationship between key age-related PI genes and drug sensitivity, we first downloaded drug-gene interactions (version 2022-Feb) from DGIdb (https://dgidb.genome.wustl.edu/) and then extracted drugs for key Age-related PI gene and TF gene interactions.

### 2.12. KEGG Pathways Enriched in Key Age-Related PI Genes

In order to analyse the biological functional relationships between key age-related PI genes and other ageing genes, we extracted the pathways of the key age-related PI gene and other ageing genes from the KEGG database (https://www.kegg.jp/). Other ageing genes included the nonkey age-related PI genes and the ageing gene from the Ageing Atlas database, with a total of 524 genes. Finally, we used Cytoscape to map the pathways of key age-related PI genes and other ageing genes.

## 3. Results

### 3.1. Differential Gene Expression Analysis

We used the “*limma*” package in R to analyse the differential expression of PI and ageing-related genes. For the PI dataset GSE33774, we selected the genes with *p* value < 0.05 and |log(fold change)| > 0.5 as differentially expressed genes, where log2(FC) > 0.5 indicated upregulated genes, and log2(FC) < −0.5 indicated downregulated genes. For the PI dataset GSE106090, we selected the genes with *p* value < 0.05 and |log(fold change)| > 1.5 as DEGs, of which those with log2(FC) > 1.5 were upregulated genes and those with log2(FC) < −1.5 were downregulated genes.

For GSE180588 and GSE83382 datasets of ageing, we selected the genes with *p* value < 0.05 and |log(fold change)| > 0.5 as DEG, where Log2(FC) > 0.5 indicated upregulated genes, and log2(FC) < −0.5 indicated downregulated genes.

**Figure 1:**
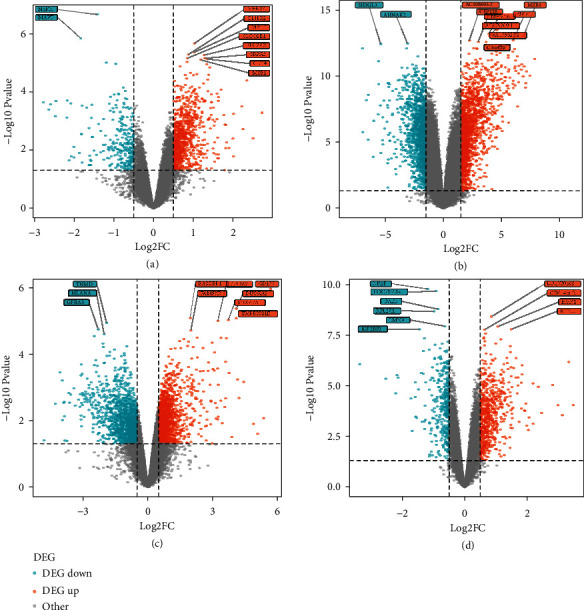
(a, b) Pi and (c, d) Age differentially expressed gene distribution volcano map.

The number of DEGs obtained is shown in Table 2. A volcano plot depicted the distribution of DEGs (Figures 1(a)–1(d)) in the four datasets, where the top 10 genes with the lowest *p* values were displayed.

### 3.2. Age-Related PI Gene Screening

**Figure 2:**
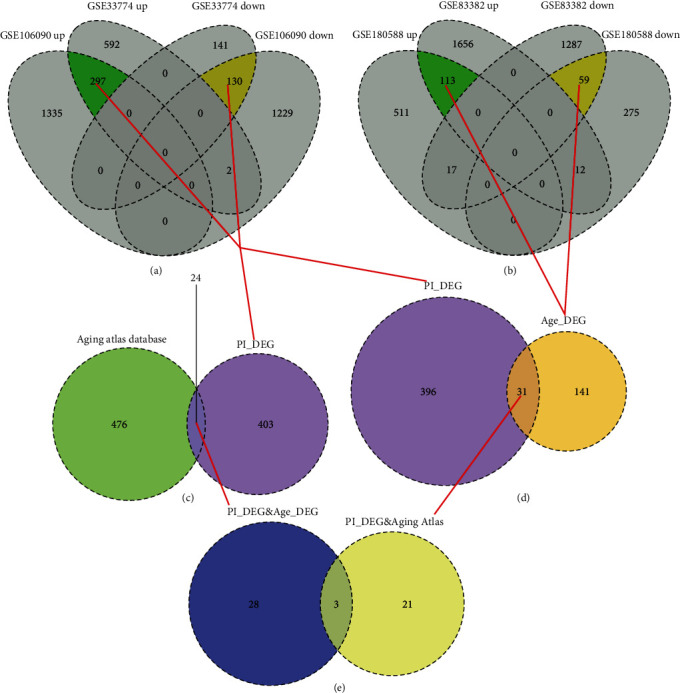
(a) Differential expression gene relationships between the two datasets of PI. A total of 427 PI differentially expressed genes were obtained, including 297 upregulated genes and 130 downregulated genes. (b) Differential expression gene relationships between the two datasets of ageing. A total of 172 PI differentially expressed genes were obtained, including 113 upregulated genes and 59 downregulated genes. (c) Venn diagram showing the intersection of PI differentially expressed genes and the Aging Atlas database. (d) Venn diagram of PI differentially expressed genes and ageing-related differentially expressed genes. (e) Venn diagram of the two age-related PI genes.

We extracted the genes that were coupregulated and codownregulated in the two datasets of PI and finally obtained 427 PI DEGs, including 297 upregulated genes and 130 downregulated genes (Figure 2(a)). We extracted the genes that were coupregulated and codownregulated in the two ageing datasets and obtained 172 ageing-related DEGs, including 113 upregulated genes and 59 downregulated genes (Figure 2(b)). We obtained a total of 31 genes that differed significantly in both PI and ageing (Figure 2(d)), labelled as age-related PI gene set 1.

We obtained a total of 500 ageing-related genes from the Ageing Atlas database, of which 24 genes showed significant differences in PI (Figure 2(c)), which were labelled age-related PI gene set 2. We combined age-related PI gene set 1 and age-related PI gene set 2 to obtain a total of 52 genes. The three genes MMP1, KCNA3, and IL1B appeared in both gene sets (Figure 2(e)).

### 3.3. Biological Functions Enriched in Age-Related PI Genes

**Figure 3:**
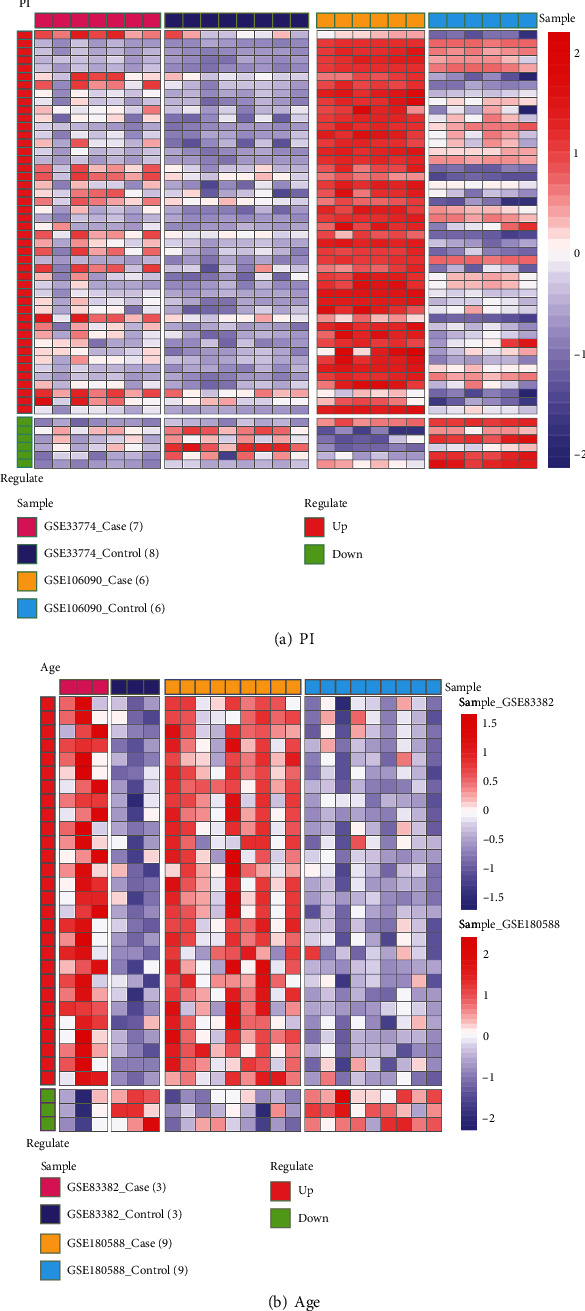
Age-related PI gene expression spectral heat map in PI (a) and age (b) datasets.

We extracted the expression values of the 52 age-related PI genes in PI datasets and used heat maps to depict gene expression (Figure 3(a)). Expression values of the 31 genes in age-related PI gene set 1 in the ageing dataset were obtained, displayed as a heat map (Figure 3(b)). From Figure 3, significant differences in age-related PI genes' expression values between the case and the control groups were evident.

**Figure 4:**
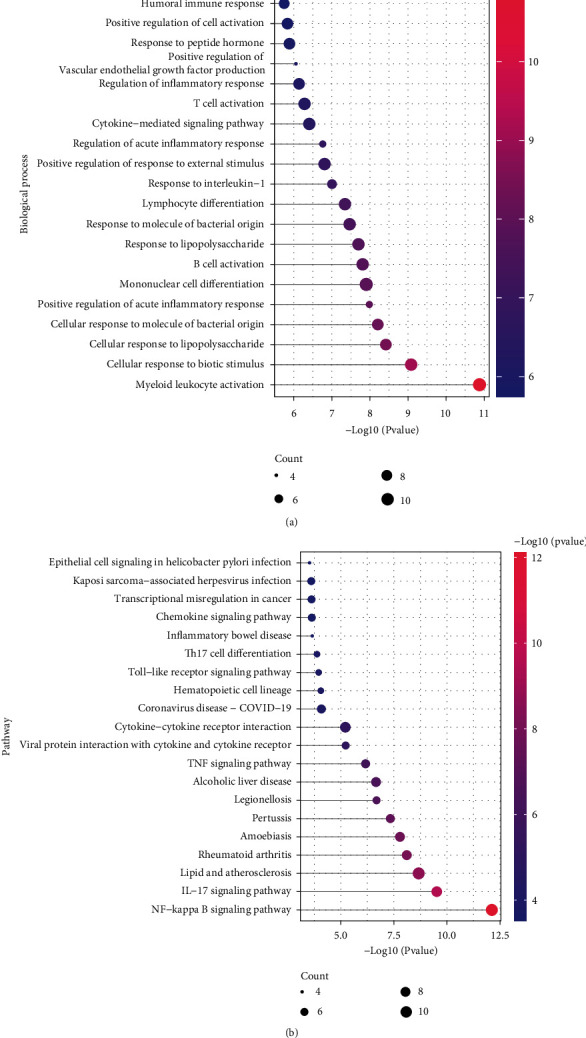
Age-related PI gene regulated functions. (a) Biological processes showing significant enrichment of age-related PI genes. (b) KEGG pathways significantly enriched in the age-related-PI genes.

We used the “*clusterProfiler*” package in R for “GO Biological process” and “KEGG pathway” analysis for the 52 age-related PI genes. We selected pathways with *p* value < 0.05 as significant and chose the top 25 pathways for presentation (Figures 4(a) and 4(b)). The results showed that the age-related PI genes mainly regulated biological processes including myeloid leukocyte activation, positive regulation of acute inflammatory response, mononuclear cell differentiation, and B cell activation (Figure 4(a)). In addition, age-related PI genes participated in NF-kappa B signalling pathway, IL-17 signalling pathway, TNF signalling pathway, and other related pathways (Figure 4(b)).

### 3.4. PPI Network of Age-Related PI Genes

**Figure 5:**
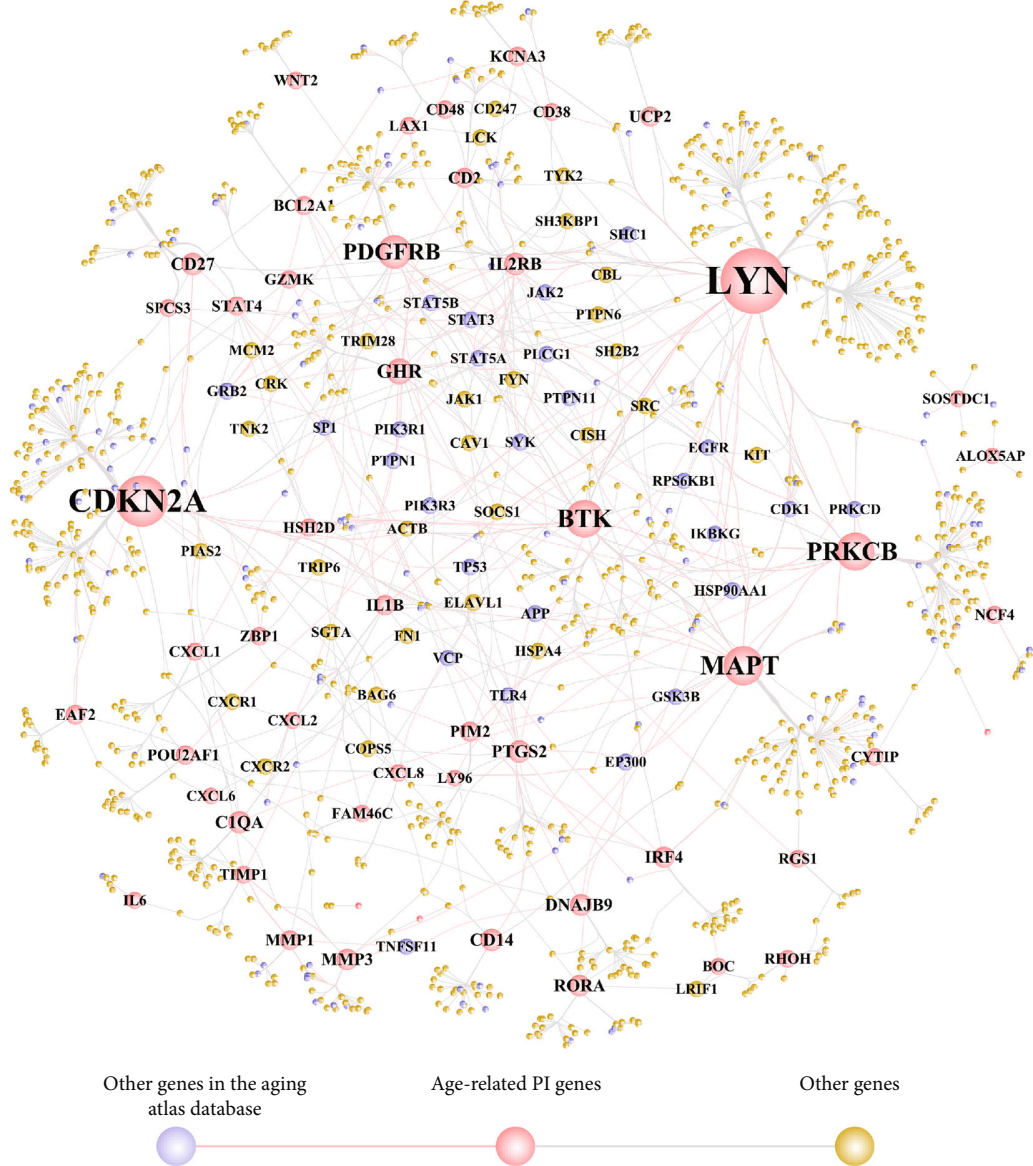
Age-related PI gene PPI network. Since there were a large number of nodes, the nodes lower in degree were hidden and nodes higher in degree were displayed. Nodes in the network include age-related PI genes and genes including other genes in the Aging Atlas database and nonageing-related genes.

We extracted protein pairs for age-related PI gene interactions from the HPRD and BIOGRID databases and then used the Cytoscape software to build a PPI network (Figure 5). The network consisted of 1172 nodes and 1441 relationship pairs.

We performed a topological property analysis of the network and then arranged the nodes in descending order of degree, filtering the top 20 (Table 3) with higher degree. As can be seen from the topological properties, LYN, CDKN2A, MAPT, BTK, and PRKCB were hub genes in the PPI network.

### 3.5. Correlation Analysis between Age-Related PI Gene

**Figure 6:**
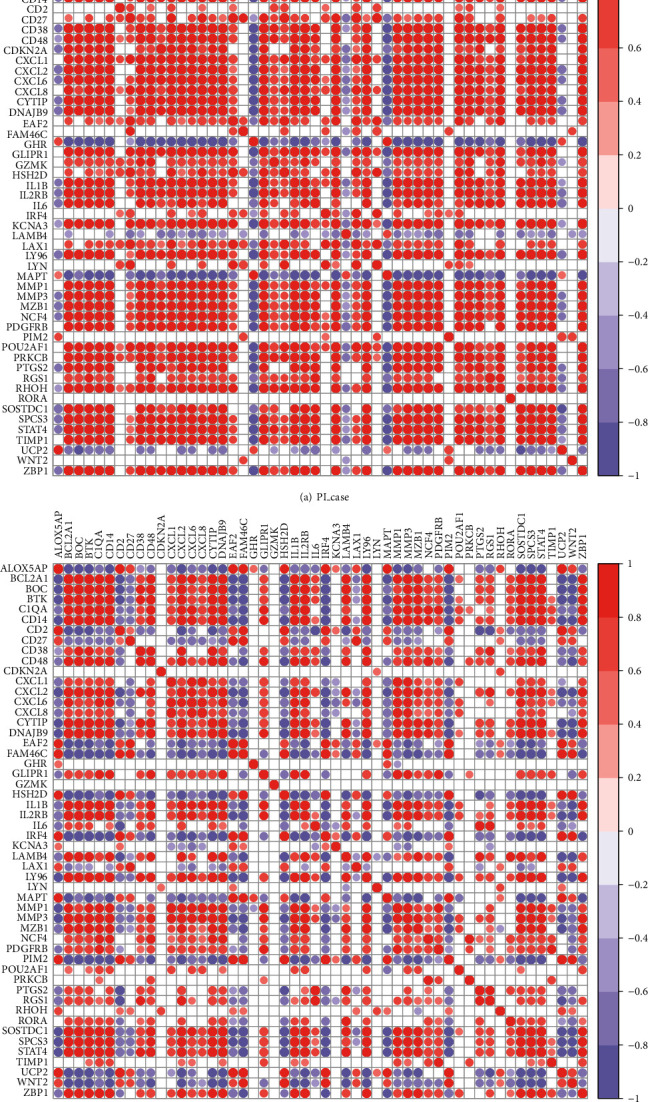
Correlation coefficient of age-related PI gene in PI case (a) and control (b). The correlation coefficient of the significance *p* value > 0.05 is hidden in the figure. The smaller the value of the test result, the more “∗” on the graph, and the correspondence between the *p* value and the “∗” sign is ns: *p* > 0.05; ∗: *p* ≤ 0.05; ∗∗: *p* ≤ 0.01; :*p* ≤ 0.001; :*p* ≤ 0.0001.

We combined the two datasets of PI to obtain a total of 13 case samples and 14 control samples. We extracted the age-related PI genes' expression values in the pooled samples, then computed Pearson's correlation coefficient to analyse the relationship between the genes, and used the R corrplot package for depiction (Figures 6(a) and 6(b)).

Based on correlation analysis, we obtained gene relationship pairs that were highly positively correlated in both case and control samples: CD14 and LY96 (cor = 0.9983, case), CD14 and DNAJB9 (cor = 0.9982, case), CD14 and BCL2A1 (cor = 0.9974, case), CD14, and BTK (cor = 0.9972, case).

### 3.6. Immune Cell Infiltration Analysis for PI

**Figure 7:**
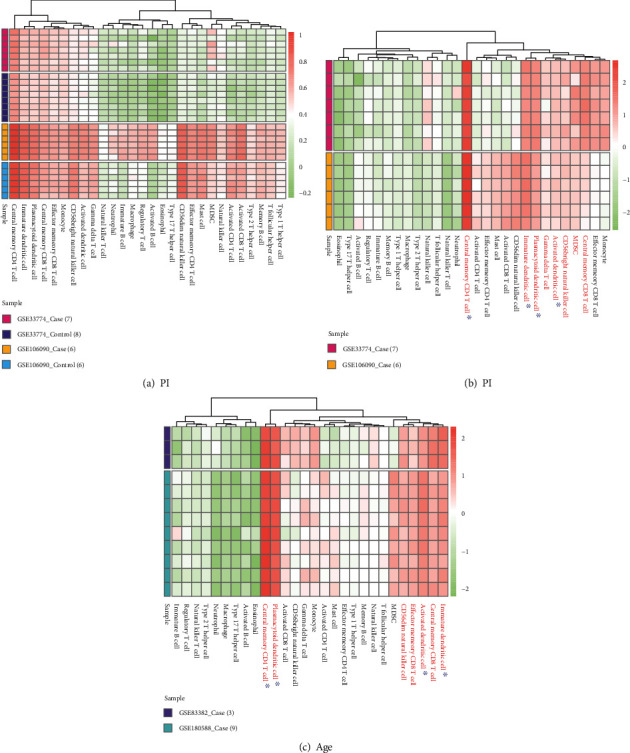
Abundance of immune cells in PI and age samples. (a) Abundance of immune cells in all samples of PI datasets (GSE33774 and GSE106090). (b) Abundance of immune cells in the case samples of PI datasets (GSE33774 and GSE106090). (c) Abundance of immune cells in the case samples of ageing datasets (GSE83382 and GSE180588).

We obtained the immune cells and gene relationships from the literature [19] and performed ssGSEA analysis for case samples of the two datasets (GSE33774 and GSE106090) of PI based on the immune cells and gene relationships. We combined the results of the two datasets' analyses and used the “*pheamap*” package to depict cellular abundance (Figure 7(a)) of these immune cells. We extracted the abundance fractions of the immune cells in the case samples and used hierarchical clustering to obtain 8 immune cells with relatively high cell abundance (Figure 7(b)). We also performed ssGSEA for case samples from the 2 datasets (GSE83382 and GSE180588) for ageing. Thereafter, we obtained 7 immune cells with relatively high abundance (Figure 7(c)). Through ssGSEA (Figure 7), we showed that activated dendritic cells, central memory CD4 T cells, immature dendritic cells, and plasmacytoid dendritic cells were highly abundant in both PI and ageing.

**Figure 8:**
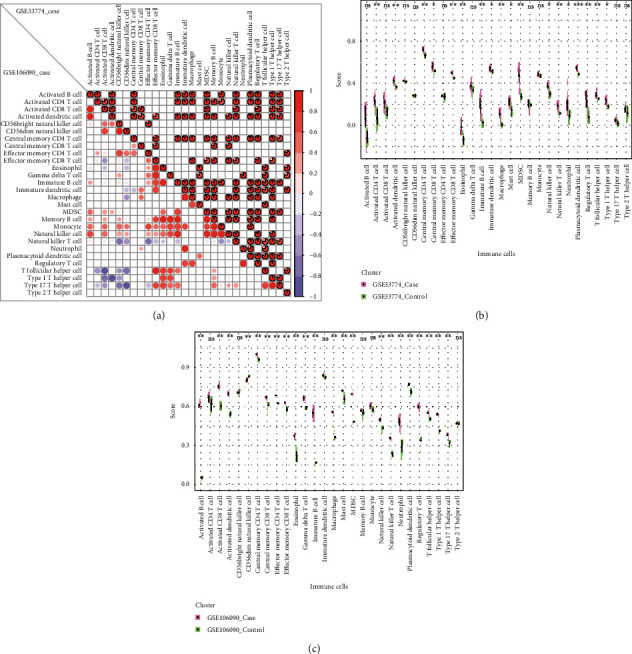
Correlation and significance analysis of immune cells in PI. (a) Correlation analysis of immune cells in PI, the lower left half is the correlation between case samples in GSE106090, and the upper right half is the correlation between case samples in GSE33774. Significant correlation coefficients (*p* value < 0.05) are shown. (b) Differential analysis of the abundance of immune cells in the PI dataset GSE33774. (c) Differential analysis of the abundance of immune cells in the PI dataset GSE106090.

Pearson correlation coefficient was computed to analyse the correlation of immune cells in the case samples of PI (GSE33774 and GSE106090) (Figure 8(a)). The results showed that the activated dendritic cell and the immersive dendritic cell were highly positively correlated in both datasets.

The abundance of immune cells in control samples and case samples of PI datasets (GSE33774 and GSE106090) was analysed for differences using the Wilcoxon test (Figures 8(b) and 8(c)). The results showed that 18 immune cells had significant differences in the dataset GSE33774, including 4 high-abundance immune cells (activated dendritic cell, central memory CD4 T cell, immature dendritic cell, and plasmacytoid dendritic cell) were significantly different (Figure 8(b)). In addition, we obtained 22 immune cells with significant differences in the dataset GSE106090, of which the immature dendritic cell did not have significant differences, and the other 3 high-abundance immune cells (activated dendritic cell, central memory CD4 T cell, and plasmacytoid dendritic cell) had significant differences (Figure 8(c)).

### 3.7. Further Screening for Key Age-Related PI Genes

**Figure 9:**
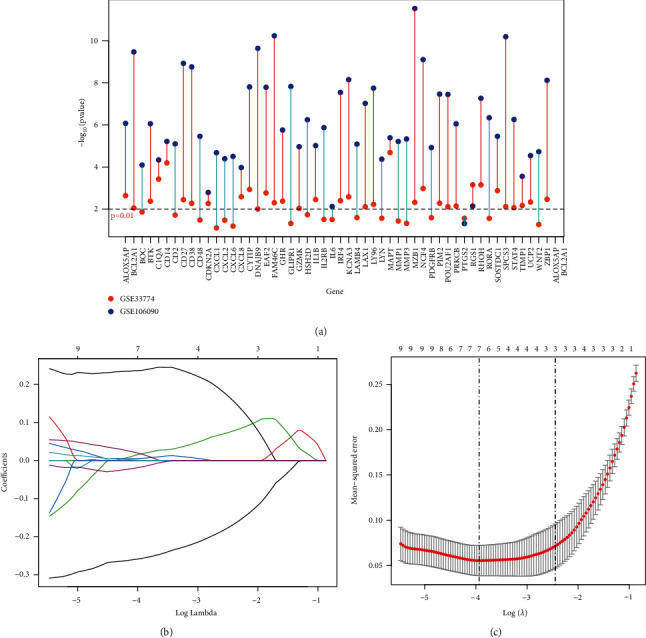
Key age-related PI genes screening. (a) ANOVA analysis of age-related PI genes. *p* value < 0.01 is a significant gene. (b) Change curves of characteristic gene. The *x*-axis shows the logarithm of the lambdas, the *y*-axis shows the variable coefficient, and the *x*-axis (above) is the remaining number of variable genes whose variable coefficient is not 0 under the log value of the current lambda. (c) Cross-checking of the lambda result. There are two dashed lines in the figure, one is lambda.min with the minimum mean square error, and the other is lambda.1se with the standard error from the minimum mean square error.

We extracted the expression values of the age-related PI genes in the PI dataset (GSE33774 and GSE106090) and then used ANOVA for variance analysis. We obtained 34 significant age-related PI genes (*p* value < 0.01 in two datasets) (Figure 9(a)) and then used LASSO logistic regression to remove redundant features of 34 age-related PI genes (Figures 9(b) and 9(c)). Finally we obtained 7 significant key age-related PI genes (ALOX5AP, EAF2, FAM46C, GZMK, MAPT, RGS1, and SOSTDC1).

**Figure 10:**
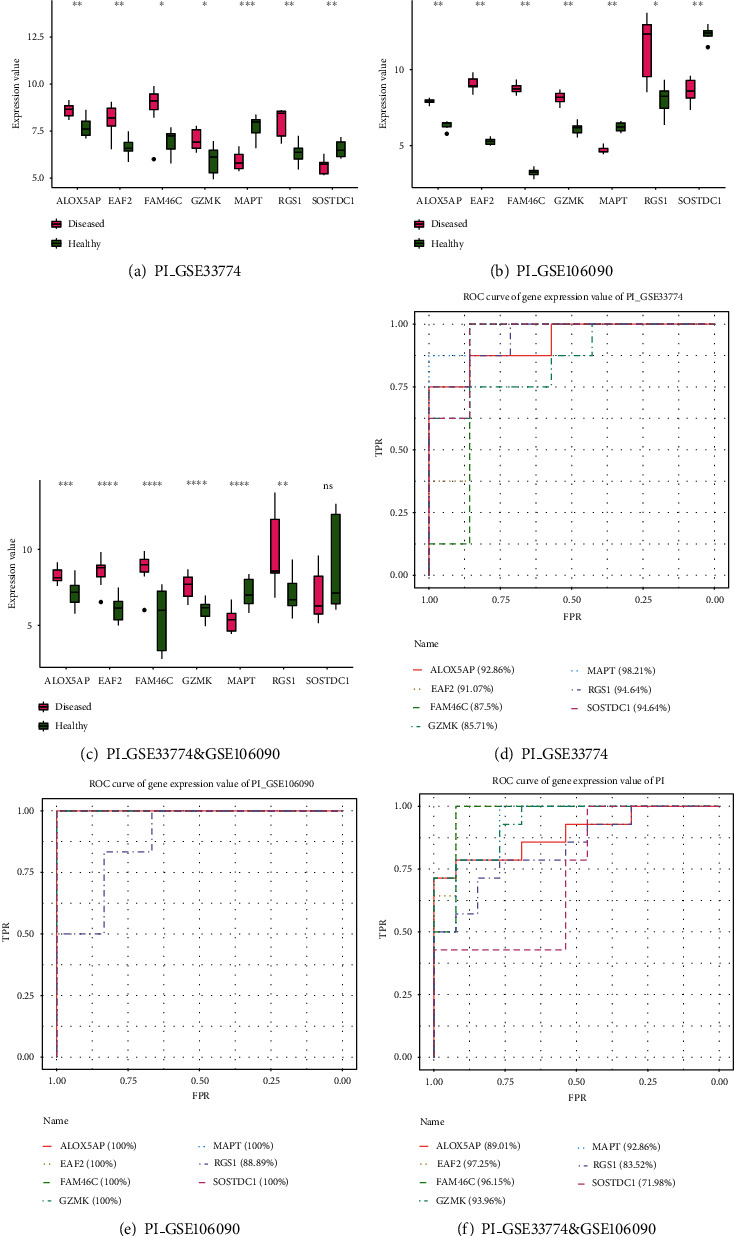
Key age-related PI gene expression levels and ROC analysis. (a) Expression of key age-related PI genes in GSE33774. (b) Expression of key age-related PI genes in GSE106090. (c) Expression of key age-related PI genes in PI combined datasets (GSE33774 and GSE106090). (d–f) ROC analysis results of key age-related PI genes in GSE33774, GSE106090, and PI combined datasets (GSE33774 and GSE106090).

We extracted the expression values of 7 key age-related PI genes from GSE33774 and GSE106090 and then applied the Wilcoxon test for difference analysis. The results showed that the 7 key age-related PI genes were differentially expressed between disease and control samples in both datasets (Figures 10(a) and 10(b)). We combined the GSE33774 and GSE106090 samples and then extracted the expression values of key age-related PI genes in the combined samples and analysed the differences. The results showed that only SOSTDC1 was not differentially expressed, and the remaining 6 key age-related PI genes were differentially expressed between case and control samples (Figure 10(c)). We performed a ROC analysis for the 7 key age-related PI genes, which key showed AUC values that were all greater than 70%, with a high predictive effect (Figures 10(d)–10(f)).

### 3.8. Relationship between High-Abundance Immune Cells and Key Age-Related PI Gene

**Figure 11:**
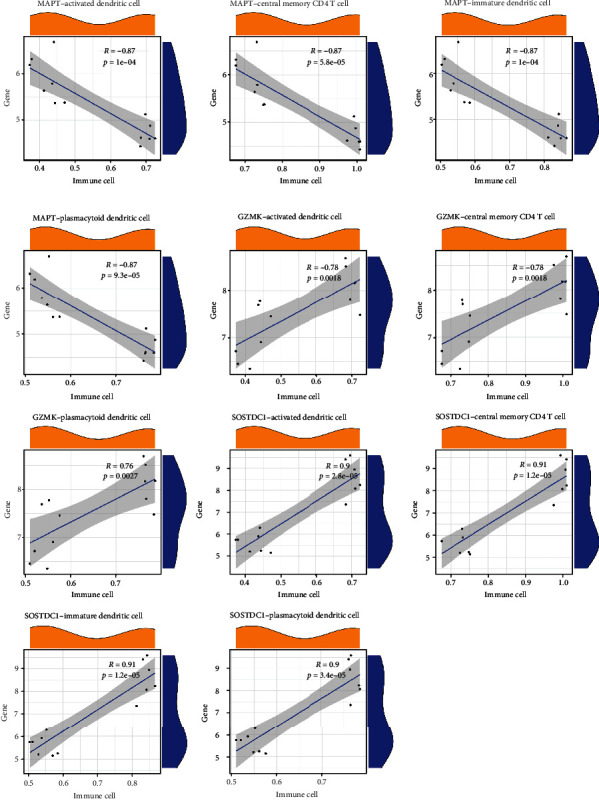
Relationship between high-abundance immune cells and key age-related PI genes.

We further analysed the relationship between immune cells and key age-related PI genes. We first obtained the abundance values of 4 high-abundance immune cells in the case samples of PI and expression values of 7 key age-related PI genes in the same samples. For each immune cell and key age-related PI gene, we performed a correlation analysis. We selected the correlation pairs with *p* value < 0.05 and |Correlation| > 0.75 in the analysis results for display. The results showed that MAPT, GZMK, and SOSTDC1 were highly correlated with high abundance of immune cells (Figure 11).

### 3.9. Key Age-Related PI Gene, Transcription Factor, and Drug Relationship

**Figure 12:**
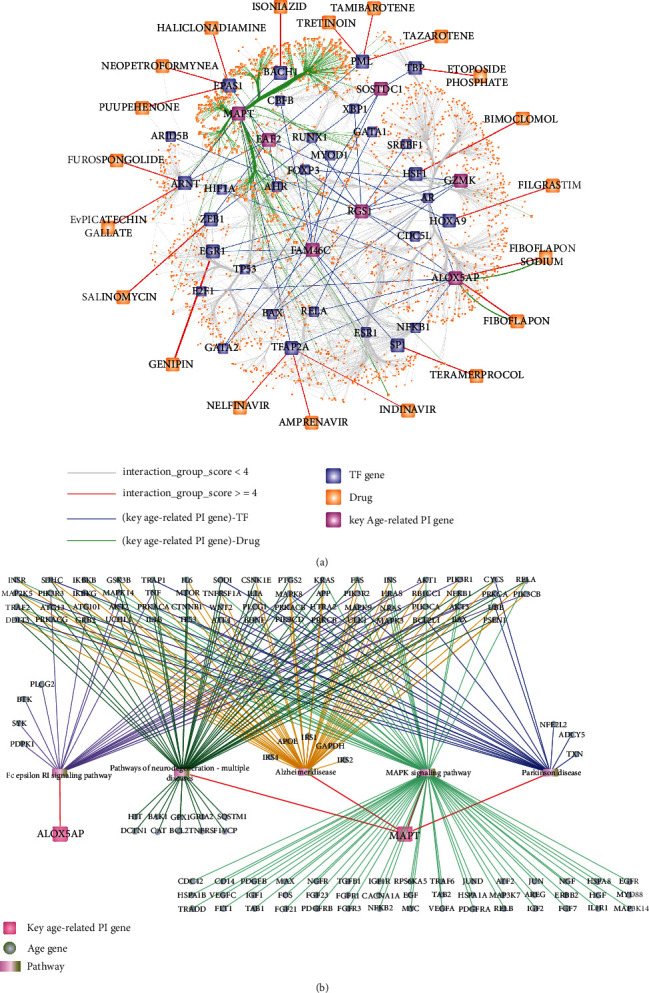
Regulatory relationship of key age-related PI gene. (a) Key age-related PI gene, TF, and drug relationship. The interaction score is a static score in DGIdb. We only show nodes with an interaction group score ≥ 4, and the other nodes are hidden. (b) Key age-related PI gene and pathway relationship.

We obtained drugs targeting key age-related PI genes from the DGIdb database, and then, we extracted key age-related PI gene and TF relationship pairs. Then, we used TF as a medium to merge the drug-target pairs and the TF-target relationship pairs. In addition, we downloaded drug-TF pairs from the DGIdb database, mining for TF regulated key age-related PI genes. Finally, the above relationship pairs were integrated to obtain drug-target-TF relationship pairs (target is key age-related PI gene). We used Cytoscape software to demonstrate the relationships among key age-related PI genes, drugs, and TFs (Figure 12(a)). The network included 1905 nodes and 2459 interrelated edges. The result showed that MAPT and ALOX5AP obtained were associated with multiple drugs and regulated by TF.

In addition, we obtained biological pathways enriched in the key age-related PI genes and other age-related genes from the KEGG database. Five enriched pathways were identified including Parkinson disease, pathways of neurodegeneration-multiple diseases, MAPK signalling pathway, Fc epsilon RI signalling pathway, and Alzheimer disease. We used Cytoscape to build pathway-gene networks (Figure 12(b)). The network includes 136 interleaved nodes and 233 edges. From the results obtained, ALOX5AP and MAPT were found to interact with other age-related genes to regulate multiple biological pathways.

## 4. Discussion

Using intersection analysis, 52 genes were identified as age-related-PI genes and found enriched in multiple inflammation-associated processes including myeloid leukocyte activation, acute inflammatory response, mononuclear cell differentiation, B cell activation, NF-kappa B signalling, IL-17 signalling, and TNF signalling. The ageing process has been associated with deregulated chronic and low-grade inflammation via mechanisms of molecular inflammation, immunosenescence, and “inflammaging” [23–25], which can exacerbate inflammatory responses to pathogenic stimuli. Increased NF-kappa B signalling with higher levels of IL-6 and TNF-*α* and related receptors is seen in aged tissues [26]. Higher C-reactive protein levels indicative of the acute inflammatory response are also evident [27]. These findings and our results together suggest that ageing-associated exacerbated inflammatory mechanisms can contribute significantly to peri-implantitis pathology in elderly subjects, as with other age-associated inflammatory and metabolic disorders [28]. Age-associated NF-kappa B signalling activation is considered a central mechanism in ageing-related inflammation and is highly responsive to redox stress [29], indicating heightened responses to plaque biofilm pathogens may occur in peri-implant tissues of aged individuals.

LYN, CDKN2A, MAPT, BTK, and PRKCB were evident as hub genes in the PPI network. These genes are important regulators of immune responses, neurodegeneration, and autophagy pathways. Lck/yes-related protein tyrosine kinase (LYN) is involved in regulation of multiple immune cells including dendritic cells, T, and B cells [30–32]. CDKN2A (cyclin dependent kinase inhibitor 2A/multiple tumor suppressor 1) gene is linked to immune infiltration in multiple cancers [33], and it regulates anti-inflammatory cytokine IL-4 and CD8+ T cell population [34]. The microtubule-associated protein Tau encoding gene MAPT, which was also identified as a key-age-related PI gene, is strongly associated with neurodegeneration and related diseases including Alzheimer's and Parkinson's diseases [35] and also associated with bone-mineral density [36]. The Bruton's tyrosine kinase (BTK) gene is an important regulator of B cell receptor signalling and innate immune cells including macrophages and dendritic cells [37]. Protein kinase C *β* (PRKCB) functions as a mitochondrial energy regulator and inhibits autophagy [38].

Multiple dendritic cell populations including activated dendritic cells, immature dendritic cells, and plasmacytoid dendritic cells along with central memory CD4 T cells were found highly abundant in PI and ageing tissues by immune cell infiltration analysis. Dendritic cell functions are impaired with age, leading to attenuated phagocytic and migratory capacity. Furthermore, aged dendritic cells produce higher proinflammatory cytokines, with lowered self-antigen tolerance and T-cell induction capacity [39], all of which may exacerbate proinflammatory responses to plaque microorganisms and also increase foreign body response to dental implant titanium [40] that can contribute to peri-implantitis. In aged individuals, the proportions of naïve CD4 T cells decline whereas memory CD4 T cells are increased [41]. A higher proportion of memory CD4+ cells in age are associated with dysregulated cytokine production, impaired T cell function and immune responses, and chronic disease occurrence [42].

7 key age-related PI genes including ALOX5AP, EAF2, FAM46C, GZMK, MAPT, RGS1, and SOSTDC1 were identified using LASSO and showed high predictive value in distinguishing cases and controls. The key age-related PI genes were linked to several immune-related functions in experimental studies including autoimmune responses. Among the key genes, MAPT and ALOX5AP were found associated with several drugs and transcription factors and also interacted with other age-related genes to regulate multiple biological pathways. The leukotriene pathway includes 5-lipoxygenase (5-LO) activating protein, encoded by ALOX5AP [43]. Leukotriene pathways are implicated in multiple ageing-related inflammatory diseases [44]. The ELL-associated factor 2 (EAF2) gene is implicated in preventing autoimmune responses by promoting B cell apoptosis [45]. The FAM46 gene is implicated in regulating T and B cells and found negatively associated with naïve CD4 + cells in pan-cancer [46] GZMK encodes Ganzyme K, a serine protease closely associated with proinflammatory responses and impediment of wound-healing by inflammation and impaired epithelialization [47]. A subpopulation of age-associated granzyme K- (GZMK-) expressing CD8+ T (Taa) cells have been identified as a source of proinflammatory granzyme K and considered a valuable target for age-linked immune dysfunction [48]. The potential role of Taa cells in aged peri-implantitis lesions is not yet experimentally investigated. RGS1 (regulator of G-protein signalling 1) is linked to multiple autoimmune disorders via control of T and B cell signalling, including T follicular helper cell population [49]. Sclerostin domain containing 1 (SOSTDC1) inhibits osteoblast differentiation by attenuating Wnt-BMP signalling [50] and regulates natural killer (NK) cells [51]. MAPT, GZMK, and SOSTDC1 were highly correlated with high abundance of immune cells, possibly reflecting immune cell perturbations accompany ageing-associated signalling pathways to play a role in peri-implantitis lesions in aged tissues.

The 7-key age-related PI genes were found markedly enriched in multiple neurodegeneration-associated pathways, Parkinson disease, pathways of neurodegeneration-multiple diseases, and Alzheimer disease, along with MAPK signalling pathway and Fc epsilon RI signalling. The loss of periodontal ligament following tooth-loss results in mechanoreceptor and neural tissue loss resulting in tactile sensation loss, whereas dental implants show “osseoperception” owing to sensory innervation at the bone-implant junction and junctional epithelium [52]. Myelinated nerve fibres have been noted in the peri-implant bone [53]. The nerve density in peri-implant bone is lower than that around teeth [54], and little is known about changes induced by peri-implant disease. Neurotrophins, neuropeptides, and nerve cells play important roles in regulating bone tissue, and most research has focused on osseointegration [55]. The contribution of age-associated activation of neurodegenerative pathways to peri-implantitis lesions in elderly is yet to be experimentally investigated. In addition, mitogen-activated protein kinase cascade (MAPK) signalling is an important regulator of cell survival and is also implicated in neurodegeneration [56] age-associated decline in tolerance of oxidative stress [57]. Fc epsilon RI signalling is a receptor for IgE [58], present on mast cells, dendritic cells, monocytes, and eosinophils, and is implicated in type I allergic responses and T-cell priming [59]. Titanium particles and cement have been observed as foreign bodies in peri-implantitis lesions [40]. It is plausible that age-associated shifts in immune cell populations and signalling bias towards proinflammatory pathways may enhance foreign body responses in peri-implantitis among aged individuals. Overall, our findings support that age-related immunosenescence and persistent chronic inflammation that impact host response to biomaterials [60] could account for peri-implantitis in aged individuals. However, as we have performed secondary analysis of available gene expression data, these findings should be verified in future using experimental models and clinical research. While it is not possible to provide causal inference from this study, the analysis provides theoretical basis for experimental and translational research investigating the identified candidate molecular mechanisms in context of host-peri-implant interaction in the backdrop of biological ageing. These perspectives can contribute to the development of improved modalities for better implant treatment outcomes in elderly patients. Notably, for the peri-implantitis dataset GSE33774, the median age was 52 (range: 38-71) years, and for the dataset, the mean reported age for GSE106090 was 55.7 ± 13.0 years. These data indicate that the population under study consisted primarily of middle-aged and elderly adults. This age distribution is representative of the dominant age groups who receive the most numbers of dental implants [61]. With increasing ageing of global populations, the proportion of elderly individuals with dental implants is increasing [62]. Further research should focus on identifying age-specific disease characteristics of peri-implantitis lesions in elderly. The major limitation is lack of experimental data verifying the candidate genes and interrelationships identified. Furthermore, the study has utilised relatively few datasets. Addition of PI datasets, especially from lesions in aged individuals, can provide greater insights into this subject.

## 5. Conclusion

A suite of bioinformatics analysis identified a 7-signature gene set highly relevant to cooccurrence of ageing and peri-implantitis associated with inflammation, auto-immunity, and neurodegeneration-related signalling. MAPT and ALOX5AP were identified as key candidate target genes for clinical translation.

## Figures and Tables

**Table 1:** PI and age sample information statistics.

	Case	Control	Platforms
GSE33774	7 (peri-implantitis patient)	8 (healthy individual)	GPL6244
GSE106090	6 (inflamed peri-implant tissue)	6 (healthy periodontal tissue)	GPL21827
GSE83382	3 (old)	3 (young)	GPL11154 (Illumina HiSeq 2000)
GSE180588	9 (gingival tissue at BL, aged NHP)	9 (gingival tissue at BL, young NHP)	GPL17015

**Table 2:** Counts of different expressed genes for PI and age.

Datasets	PI		Age	
	GSE33774	GSE106090	GSE83382	GSE180588
*p* value	*p* < 0.05		*p* < 0.05	
|Log2(FC)|	Value > 0.5	Value > 1.5	Value > 0.5	
DEG up	271	1632	1781	641
DEG down	891	1361	1363	346
Total DEG	1162	2993	3144	987

**Table 3:** The topological characteristic of top 20 age-related PI gene in PPI networks.

Symbol	Degree	ASPL	BC	CC	TC
LYN	222	2.660606	0.39286	0.375854	0.006757
CDKN2A	159	3.135931	0.252187	0.318885	0.016009
MAPT	106	3.129004	0.178415	0.31959	0.027576
PRKCB	100	2.85974	0.191758	0.349682	0.012247
BTK	98	2.850216	0.178826	0.350851	0.013164
PDGFRB	80	3.347186	0.104298	0.298758	0.05
GHR	46	3.733333	0.046809	0.267857	0.072011
IL2RB	31	3.203463	0.036329	0.312162	0.038519
CD27	31	3.835498	0.048607	0.260722	0.037634
C1QA	30	3.916017	0.045632	0.255361	0.058333
CD14	30	4.263203	0.042613	0.234565	0.046667
PTGS2	30	3.361039	0.060437	0.297527	0.058333
DNAJB9	28	4.119481	0.0458	0.242749	0.043651
RORA	27	3.986147	0.04784	0.250869	0.045267
MMP3	26	4.594805	0.03939	0.217637	0.057692
IRF4	25	3.998268	0.032101	0.250108	0.06
CD2	21	3.663203	0.024086	0.272985	0.076605
IL1B	21	3.116017	0.057988	0.320922	0.049829
PIM2	20	3.693506	0.027603	0.270745	0.07
UCP2	19	4.522944	0.025891	0.221095	0.105263

## Data Availability

The datasets used and/or analysed during the current study are available from the corresponding author on reasonable request.
